# Continuous-wave highly-efficient low-divergence terahertz wire lasers

**DOI:** 10.1038/s41467-018-03440-4

**Published:** 2018-03-16

**Authors:** Simone Biasco, Katia Garrasi, Fabrizio Castellano, Lianhe Li, Harvey E. Beere, David A. Ritchie, Edmund H. Linfield, A. Giles Davies, Miriam S. Vitiello

**Affiliations:** 1 NEST, CNR—Istituto Nanoscienze and Scuola Normale Superiore, Piazza San Silvestro 12, 56127 Pisa, Italy; 2 School of Electronic and Electrical Engineering, University of Leeds, Leeds, LS2 9JT UK; 3 Cavendish Laboratory, University of Cambridge, Cambridge, CB3 0HE UK

## Abstract

Terahertz (THz) quantum cascade lasers (QCLs) have undergone rapid development since their demonstration, showing high power, broad-tunability, quantum-limited linewidth, and ultra-broadband gain. Typically, to address applications needs, continuous-wave (CW) operation, low-divergent beam profiles and fine spectral control of the emitted radiation, are required. This, however, is very difficult to achieve in practice. Lithographic patterning has been extensively used to this purpose (via distributed feedback (DFB), photonic crystals or microcavities), to optimize either the beam divergence or the emission frequency, or, both of them simultaneously, in third-order DFBs, via a demanding fabrication procedure that precisely constrains the mode index to 3. Here, we demonstrate wire DFB THz QCLs, in which feedback is provided by a sinusoidal corrugation of the cavity, defining the frequency, while light extraction is ensured by an array of surface holes. This new architecture, extendable to a broad range of far-infrared frequencies, has led to the achievement of low-divergent beams (10°), single-mode emission, high slope efficiencies (250 mW/A), and stable CW operation.

## Abstract (web summary)

Quantum cascade lasers are compact sources, but simultaneously achieving cw operation, low divergence and single-mode emission has proven difficult. Here, Biasco et al. use a combination of distributed feedback and outcoupling via hole arrays, improving the performance of such a terahertz laser.

## Introduction

Terahertz (THz) frequency technology has stimulated a major surge in interdisciplinary research over the last decade, inspiring both fundamental insights and new applications in microscopic^1^ and macroscopic systems^2^, as well as highlighting potential new research avenues in the fields of biomedical imaging, astronomy, security, and high-resolution sensing, for example. The quantum cascade laser (QCL)^3^ has been central to this, and has elevated THz photonics to a new level of performance, and possibility. The groundbreaking QCL design, which exploits intersubband transitions between electronic wave functions engineered on a nanometer scale, enables the optical dispersion, carrier transport and gain spectrum to be tailored, allowing a wealth of unique physical properties and operational characteristics to be achieved.

The development of modern nanofabrication technologies, combined with new laser resonator concepts, have recently enabled the control and confinement of electron and photon paths in optoelectronic devices with an unprecedented degree of control. For example, microcavities^4^, photonic crystals^5,6^, and both pseudo-random^7,8^ and random^9^ photonic structures, can manipulate and confine light in small volumes, and at targeted frequencies. This has further expanded the functionality of the THz QCL, allowing operation at a single emission frequency^7^ or over a broad (0.5 THz) frequency bandwidth, or with designed directional beam patterns^8^.

These approaches rely on lithographic patterning of the top metal waveguide surface, which enables the guided mode to be matched to the externally propagating photons, generating a collimated beam from the laser surface by interference. In a one-dimensional periodic structure with surface periodicity *Λ*, light extraction can be tailored on the fundamental (*m* = 1) spatial harmonic *k*_1_ = 1/*Λ* of the grating, while optical feedback inside the resonator is provided by a wavevector with *k*_*m*_ = *m*/*Λ*^10^. Based on the order *m* of the feedback wavevector, one-dimensional distributed feedback (DFB) structures^11–13^ can be engineered to provide directional emission over a specific plane, governed by the interaction between the light wavevector and the feedback wavevector^14^.

In these architectures, high-power emission, beam shaping, and mode control are related to the extraction of symmetric and anti-symmetric resonant eigenmodes. In general, symmetric modes produce constructive interference in the far-field and have efficient outcoupling into free space. In contrast, anti-symmetric modes interfere destructively in the far-field, but have lower loss. As such, anti-symmetric modes are favored for lasing, but consequently provide limited extraction power. A number of photonic approaches have been explored to circumvent these intrinsic limitations, while still ensuring directional beam profiles. These include vertically emitting graded photonic heterostructures^15,16^, quasi-periodic gratings^17^, and double-periodicity DFB gratings, engineered to achieve a simultaneous tailoring of the emission frequency and a tuning of the beam direction, via the independent control of the extraction and feedback wavevectors^18^. Further approaches have utilized on-chip phased locked arrays^19,20^ and metasurface reflectors, which comprise multiple cavities^21^, and induce directional THz QCL emission in pulsed operation.

In contrast to surface-emitting approaches, edge-emitting structures with narrow cavity widths are potentially more suited for applications in sensing, spectroscopy or metrology due to the lower electrical power dissipation, which better enables continuous-wave (CW) operation. In-plane emitting wire lasers exploiting third-order DFB gratings^20,22,23^, and including integrated micro-antennas^24^, are the most exploited solutions in this respect. Despite the clear advantages in the emission profiles (10° divergence)^20,22^ and slope efficiencies (120 mW A^−1^) these approaches show a number of challenges. In particular, these devices require sophisticated waveguide engineering that constrains the effective mode index to *n* = 3, i.e., the grating order, but this is significantly smaller than that of the GaAs/AlGaAs active region (*n* = 3.56). The lithographic phase matching procedure then becomes demanding if a repeatable and robust fabrication process is to be realized. Alternatively, in-plane emitting antenna-feedback plasmonic lasers^25^ have been recently proposed to deliver good beam shaping (4° divergence), but these have limited power extraction (1–2 mW) in pulsed operation.

In this work, we demonstrate double-metal waveguided DFB THz quantum cascade wire lasers, exploiting an innovative approach in which feedback is provided by a lateral sinusoidal ridge corrugation, while light extraction is separately controlled by a hole array in the top metallization. In this case, the periodicity of the array of surface holes is not an integer multiple of the lateral corrugation controlling the feedback. The feedback grating selects the lasing frequencies and allows robust single-mode emission, exploiting the inherently high spectral purity of THz QCLs^26^, while the extraction array is finely tuned to optimize the radiation outcoupling. This architecture thereby simultaneously addresses the challenges of low-divergence, single-mode emission, high power, very good slope efficiency, also in CW regime.

## Results

### Device concept and architecture

**Fig. 1:**
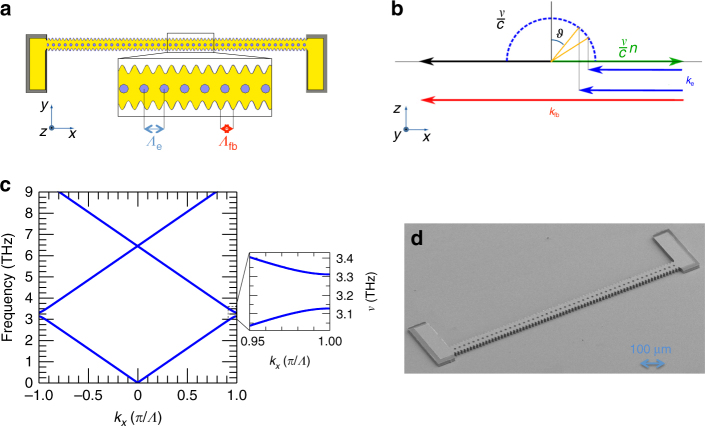
Resonator design and rationale. **a** Schematic diagrams of the laterally corrugated wire laser with a periodic (period *Λ*_fb_ = 13.6 µm) sinusoidal corrugation. The double-metal cavity comprises a central corrugated region, with two Cr pads at the ends of the resonator cavity being used to suppress unwanted electromagnetic modes. The inset shows the top metal contact (yellow) patterned with etched air holes (blue) with a periodicity *Λ*_e_, which define the extraction wavevector *k*_e_. *Λ*_e_ was varied in the range 19.4–23.7 µm, around the ideal theoretical value of 21.2 µm for an expected emission frequency of 3.1 THz. The thin Cr absorbing layer is marked in gray. **b** Light cone diagram. The feedback wavevector *k*_fb_ (red line) and the extraction one *k*_e_ (blue line) are indicated on the graph, together with the backward-propagating  (black line) and forward-propagating (green line) laser cavity wavevectors. For a given frequency *ν*, different *η* parameters provide distinct extraction wavevectors, which determine the light extraction efficiency and emission angle *ϑ* into free space (dotted blue line). Here *n* is the refractive index and *c* is the light speed in vacuum. **c** One-dimensional photonic band structure, computed on the feedback geometry only, based on a sinusoidal spatial profile of the dielectric function, and for a ridge having average width of 40 µm, a lateral corrugation length of *Λ*_fb_ = 13.6 µm, and a corrugation amplitude of 10 µm on each side of the mesa. **d** Scanning electron microscope image of a fabricated device, with hole radius, *r* = 5 µm, *Λ*_e_ = 22.4 µm (*η* = 0.95), and average width of 40 µm

Our photonic wire design was conceived to provide independent control of the feedback and extraction mechanisms through use of two independent photonic geometries. To ensure the necessary feedback for radiation propagating in the resonator, a one-dimensional photonic grating is implemented by defining a lateral sinusoidal corrugation, which modulates the width of the 10-μm-thick double-metal QCL cavity (Fig. 1a) in the 30–50 µm range. Consequently, the resonator width is comparable to the typical THz wavelength in a GaAs/AlGaAs-based QCL material (~30 µm for 3 THz radiation), making the system an almost perfect one-dimensional photonic structure. The spatial Fourier transform of the corrugation periodicity *Λ*_fb_ (Fig. 1a) defines a feedback wavevector, which is twice the light wavevector in the active material. Setting the emission frequency to 3.1 THz, the feedback wavevector *k*_fb_ = 2*n*_eff_*ν*/*c* (with *c* the speed of light in vacuum and *ν* the desired laser frequency) corresponds to a design corrugation periodicity *Λ*_fb_ = 13.6 µm, assuming an effective refractive index of the active region metallic waveguide *n*_eff_ = 3.53, as derived from the simulation of the transverse propagation of the fundamental mode in a two-dimensional slab^27^.

Light extraction into free space is controlled by a second grating: an array of circular holes lithographically defined on the top surface of the laser. The underlying semiconductor heterostructure is left exposed after removal of the upper metallic waveguide, and also the n^+^ top contact layer (by dry-etching) to prevent detrimental absorption of the outgoing radiation (Methods section). The surface holes have a diameter of 10 µm (Fig. 1a) while their periodicity, *Λ*_e_, is designed to induce a longitudinal outcoupling of the radiation with an emission angle *ϑ* = 90° with respect to the surface normal (Fig. 1b). The one-dimensional light cone diagram in Fig. 1b provides a simple description of the scattering between the light, the feedback and the extraction wavevectors.

When a guided mode propagates inside the resonator with wavevector *n*_eff_ *k*_0_ (black arrow in Fig. 1b), it is scattered by the feedback vector *k*_fb_ (red arrow in Fig. 1b) in the opposite direction (green arrow in Fig. 1b). It can further scatter with the extraction wavevector *k*_e_, which is defined via the choice hole array periodicity. By carefully tuning *k*_e_, the resulting diffracted mode can match the light wavevector in air, meaning that the vector sum of the three wavevectors must intercept the free-space light cone (blue dashed circular line, Fig. 1b) with radius *k*_0_ = *ν/c* at its bottom corner. Consequently, the value of the extraction wavevector (Methods section), required for longitudinal emission is given by:

1 $$k_{\rm {e}} = \frac{\nu }{c}\left( {n_{\rm {eff}} + \sin \,\vartheta } \right) = \frac{\nu }{c}\left( {n_{\rm {eff}} + 1} \right)$$

which determines the scattering matching condition and the periodicity *Λ*_e_. By using the parameters introduced earlier, the extraction periodicity was set to *Λ*_e_ = 21.2 µm.

Since the frequency-dependent effective refractive index can be engineered by design in a QCL, we can obtain a perfect matching condition by tuning the extraction wavevector, by spanning a 10% range around the light cone edge. To achieve this, a series of wire lasers incorporating surface hole array of different periodicities were lithographically defined to achieve a sequence of different extraction wavevectors *ηk*_e_ (with *η* in the range 0.9 < *η* < 1.1, in steps of Δ*η* = 0.05). This allowed the investigation of the optimal scattering condition, for which light extraction is enhanced and emitted in the desired direction, with low-divergence and large quantum efficiency.

The resonator geometry is characterized by two independent periodicities. The classical discrete spatial invariance of a photonic crystal is broken and a properly defined band structure no longer exists. Nonetheless, a one-dimensional model of the narrow lateral corrugation grating gives understanding of the feedback mechanism and how it selects the band-edge modes that are typically involved in lasing^28^. The spatial modulation of the dielectric function can be defined as: $\varepsilon \left( x \right) = \varepsilon _0 + \varepsilon _{\rm {d}}\,\sin \,2\pi x/ \Lambda _{\rm {fb}}$, where *ε*_0_ is the average value of the dielectric constant in the device and *ε*_d_ is the modulation associated with the sinusoidal corrugation of the ridge width. The band structure was modeled using Maxwell’s equations, and solved numerically with a Matlab code. Moreover, the upper and lower band-edge frequencies were retrieved with a perturbative approach at the edge of the Brillouin zone, i.e., around *k*_*x*_ = *π*/*Λ*_fb_, so that the center frequency *ν*_m_ is proportional to *ε*_0_^−1/2^. The relative bandgap (Δ*ν*) varies with the modulation amplitude as Δ*ν*/*ν*_m_ = *ε*_d_/2*ε*_0_, indicating that the stronger the feedback, the larger the bandgap.

Simulations performed for a TM-polarized electric field, as required by the QCL intersubband transition selection rules, show the opening of a fundamental photonic bandgap between 3.12 THz and 3.31 THz for a 40 µm ridge exploiting a sinusoidal width modulation of ±10 µm and a period *Λ*_fb_ = 13.6 µm (Fig. 1c). The average value of the dielectric constant was set to *ε*_0_ = 11.74 with a modulation amplitude *ε*_d_ = 1.34 for the fundamental mode, based on the results of the three-dimensional simulations detailed in the following section. The corresponding effective refractive index was therefore varied in the range 3.23 < *n*_eff_ < 3.62, which is roughly comparable to the value used to estimate the feedback and extraction wavevectors earlier.

Figure 1d shows a scanning electron microscope image of a typical device, devised according to the proposed architecture. The resonator consists of a sinusoidal corrugated mesa (1 mm long, 74 periods) with a pattern (42–52 periods, depending on the extraction parameter *η*) of etched extraction holes on the top metallization and two lateral rectangular pads, needed to define the electrical contacts and to provide a smooth absorbing boundary condition by the deposition of a lossy layer of chromium to suppress unwanted peripheral modes (Methods section)^7^.

**Fig. 2:**
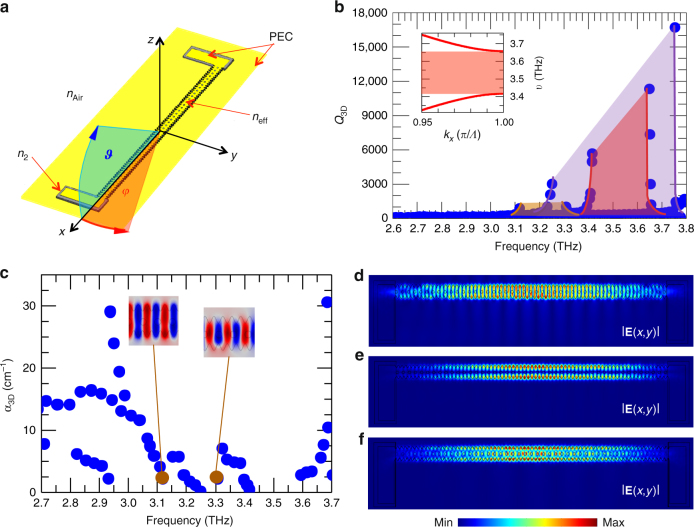
Three-dimensional simulations. **a** Schematic diagrams of the simulation geometry. The bottom substrate and the top metallic surface were simulated as perfect electric conductors (PEC) to mimic the metal layers. The active material was described by the refractive index *n*_eff_ = 3.50, the Cr pads with *n*_2_ = 4.43 + 0.31*i*, while the open boundaries outside the mesa were modeled with an external region of air (*n*_Air_ = 1), surrounded by appropriate scattering boundary conditions. According to the defined reference system, the vertical and horizontal angles of emission are *ϑ* (blue shade), lying in the *x*–*z* plane, and *φ* (red shade), lying in the *x*–*y* plane. **b** Three-dimensional quality factors, Q_3D_, computed at different eigenfrequencies for a sinusoidally corrugated resonator with a feedback periodicity *Λ*_fb_ = 13.6 µm, an average width of 40 µm, and a corrugation amplitude of 10 µm. The extraction hole pattern is neglected. The different photonic bandgaps associated with the fundamental mode (brown), the first-order lateral mode (magenta), and the second-order lateral mode (red), are shown. Inset: computed bandgap of the second-order lateral mode. **c** Total losses *α*_3D_ plotted as a function of the resonant frequencies for a 40-µm-wide wire cavity with *η* = 0.95, computed for a patterned surface array of circular air holes with periodicity *Λ*_e_ = 22.4 µm and radius *r* = 5 µm. Insets: real part of the electric field component *E*_z_ (*x,y*) for the fundamental band-edge modes above and below the bandgap. Red (blue) indicates negative (positive) values of *E*_z_. **d**–**f** Spatial distributions of the normalized electric field |*E*(*x,y*)| of the lower-edge states at half height (*z* = 5 µm) of the sinusoidal wire laser cavity. **d** is the fundamental mode at *ν* = 3.12 THz, **e** is the first-order lateral mode at *ν* = 3.25 THz, and **f** is the second-order lateral mode at *ν* = 3.42 THz

In order to simulate the lasing resonator structure robustly, including the effects of the extraction holes array and the lateral pads, a fully vectorial, three-dimensional (3D) model was implemented in a commercial (Comsol Multiphysics) finite element method (FEM) solver to study the resonating electromagnetic modes (see Methods section and Fig. 2a for device geometry). Initially, a simulation was performed of the resonator without the extraction hole array to calculate the quality factors, *Q*_3D_, and the eigenmode frequencies, dictated by the feedback lateral corrugation, in the spectral range 2.6–3.8 THz.

By comparing the electric field distributions associated with the highest *Q*_3D_, we can distinguish a set of modes with distinctive envelopes that allows the bandgaps associated with the fundamental and higher-order lateral modes to be unambiguously determined (Fig. 2b). Since the electric fields of the higher-order lateral modes typically have a larger overlap with the corrugated edges than the fundamental mode, they experience a stronger variation in refractive index, which consequently results in a larger bandgap. Indeed, the fundamental mode is centered at ~3.21 THz and has a bandgap of ∼190 GHz (~6% of the center frequency), while the first-order lateral mode has a center frequency of 3.51 THz and a bandgap of nearly 500 GHz (~14% of the center frequency). The second-order lateral mode conversely has a bandgap of ∼240 GHz, with a center frequency of 3.54 THz, which is well reproduced in the one-dimensional model for the photonic band structure with the appropriate optical constants *ε*_0_ = 9.74 and *ε*_d_ = 1.33 (see inset to Fig. 2b). By including the extraction hole array on the top metallization, the global quality factor *Q*_3D_ decreases as a consequence of the increased radiative losses for the modes with larger overlap with the patterned surface. A careful analysis of the total losses *α*_3D_ in the whole structure (Fig. 2c), for a resonator with average width 40 µm and *η = *0.95, shows that the fundamental and higher-order modes are perturbed differently. The states at the fundamental upper and lower band edges show comparable losses of about 2.5 cm^−1^ and are only slightly altered by the extraction holes (insets to Fig. 2c).

It is worth mentioning that for the case of the higher-order bandgaps, the upper-edge mode losses are instead significantly increased by the hole patterning, being five times larger than the losses of the corresponding lower-edge states, which have *α*_3D_ < 1 cm^−1^. Figure 2d, e, f show the electric field for the fundamental and higher-order modes located below the respective bandgaps, in the presence of an extraction hole array with *η = *0.95. The first-order lateral lower-edge mode is less affected by the presence of the holes, as can be easily seen by the distribution of the electric field which vanishes at the center of the mesa (Fig. 2c). The fundamental and second-order lateral modes have high-electric field intensities at the center of the bare feedback resonator, so the addition of the hole array induces distortions in the field distributions and enhances their outcoupling into free space (Fig. 2c). The 3D simulations furthermore indicate that the eigenfrequencies near the band-edge have a slight dependence on the extraction parameter *η*, since they are mainly determined by the feedback geometry.

### Transport and optical characterization

**Fig. 3:**
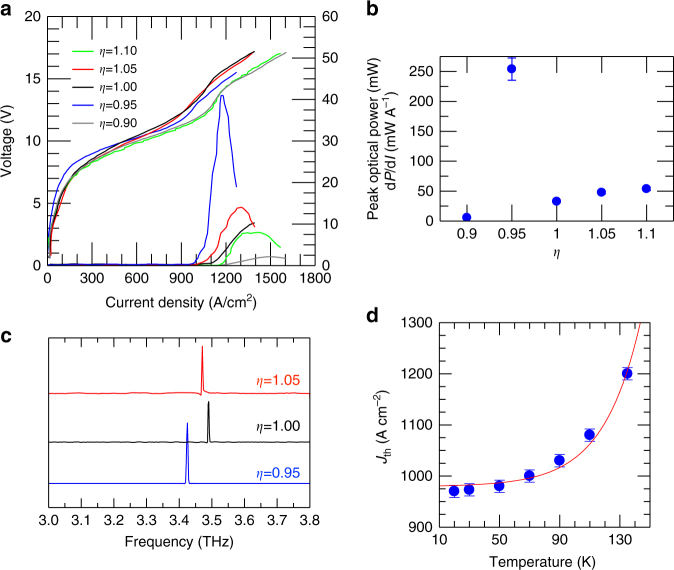
Electrical and optical characterization. **a** Voltage–current density (*V*–*J*) and power–current density (*L*–*J*) characteristics measured at 20 K for a set of devices with different *η* values, in a nitrogen-purged atmosphere. All lasers are driven in pulsed mode with a pulse width of 200 ns and pulse repetition rate of 100 kHz. Optical power scales were corrected to account for the detector collection efficiency and the absorption of the polyethylene cryostat window. The device area is 0.074 mm^2^. **b** Slope efficiency measured at 20 K plotted as a function of *η*, under the same experimental conditions shown in **a**, with error bars derived from the linear fitting procedure of the slope. **c** Fourier transform infrared emission spectra for selected devices from **a**, measured in rapid scan mode. **d** Threshold current density of the device with *η* = 0.95 as a function of the heat-sink temperature. The red line is the fit based on the empirical formula *J*_th_ (*T*) = *J*_1_ + *J*_2_ exp(*T*/*T*_2_). The error bars account for errors on the extrapolated threshold current densities

**Fig. 4:**
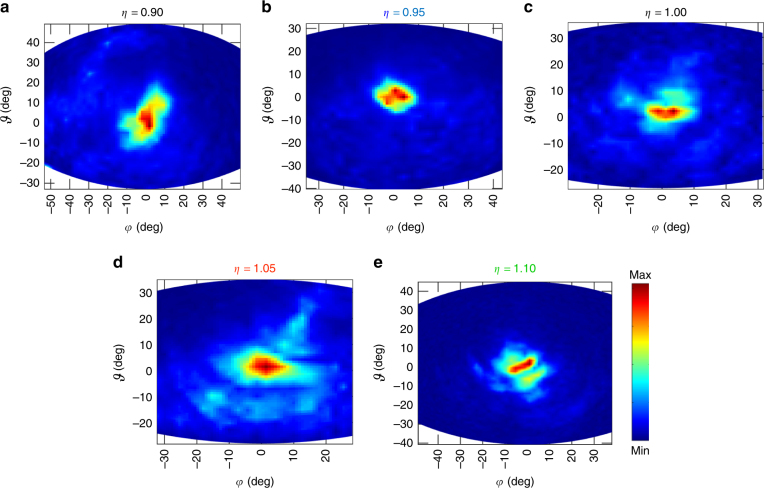
Far-field intensity patterns. Far-field emission patterns measured at 20 K while driving the devices in pulsed mode with 2% duty cycle, for wire lasers having different extraction parameters: **a** *η* = 0.90, **b** *η* = 0.95, **c ***η* = 1.00, **d** *η* = 1.05, **e** *η* = 1.10. All the measurements were performed scanning a pyroelectric detector at a distance of ~8 cm from the device lateral surface. The origin (0,0) of the plots is coincident with the origin of the *x*–*y*–*z* reference system of Fig. 2a. The scanned area is 12×12 cm

THz wire lasers (with different *η*) were initially fabricated using a broadband resonant-phonon QCL active region design^29^, with gain in the 3.0–3.7 THz range (Methods section); this provided a sufficiently wide flexibility in tuning the central frequency. Figure 3a shows the measured current density–voltage (*J*–*V*) and power–current density (*L*–*J*) characteristics of a set of five wire lasers, driven in pulsed operation with a repetition rate of 100 kHz and a pulse width of 200 ns, at a temperature of 20 K in a nitrogen-purged environment. Most lasers produce a maximum peak optical power in the range 5–15 mW. However, when the perfect matching condition is reached, here for an extraction parameter *η* = 0.95, the output power exceeds 40 mW, due to the combined effect of the enhanced coupling losses and the significant beam reshaping (Fig. 4b) which has a positive impact on the collection efficiency.

The highest optical power is also reflected in the lowest threshold current density *J*_th_ = 970 A cm^−2^, with *J*_th_ ranging between 1.0 and 1.2 kA cm^−2^ for all other extraction geometries (different *η* values). It is worth mentioning that the measured *J*_th_ in our wire lasers is comparable or slightly larger than that measured in standard Fabry–Perot (FP) cavities (~1 kA cm^−2^ for the same epitaxial material)^29^. The slope efficiency (Fig. 3b) of the *η* = 0.95 wire laser also reaches a record value of ≈250 mW A^−1^, significantly larger than that achieved (5–60 mW A^−1^) when the extraction parameters are not properly matched, and much larger than the best third-order DFB THz QCL resonators reported to date (130–140 mW A^−1^)^13,20^. Another important figure of merit for lasers is the wall-plug efficiency (WPE), which is a quantitative measure used for comparing different devices. The best *η* = 0.95 wire laser has a 0.3% WPE, a factor of three larger than corresponding FP double-metal cavities fabricated with the same double-metal sequence and without removing the highly doped top contact.

Figure 3c shows the Fourier transform infrared (FTIR) emission spectra measured at a heat-sink temperature of 10 K, while driving the QCLs in pulsed mode with a 2% duty cycle. In all cases, a single spectral line is observed across the entire QCL gain bandwidth, with its frequency shifting slightly between 3.43 and 3.49 THz when the extraction parameter is tuned over the selected range. This is in agreement with the simulation results, which identifies the lower band-edge mode of the second-order lateral excitation as being 3.42 THz for *η* = 0.95. The variation of threshold current with temperature is presented in Fig. 3d, with devices operating up to a heat-sink temperature of 135 K, which corresponds to an estimated lattice temperature *T*_L_ ≈ 144 K^30^.

In order to understand the role of the extraction hole array on the far-field beam pattern, the intensity distribution of each device was sampled by scanning a pyroelectric detector in the plane orthogonal to the *y*–*z* surface at a distance of ~8 cm. As expected, the far-field intensity plots show different divergences as a function of the extraction wavevector (Fig. 4a–e). For the case of *η* = 0.95, the beam is single-lobed and localized within a beam profile of Δ*φ* ≈ Δ*ϑ* ≈ 10° (Fig. 4b).

Such a divergence is expected to be reduced by the increase of the cavity length^20,22^, which can also have a beneficial effect on the optical power output^22^ and slope efficiency.

Our architecture also offers the potential for high power, low-divergent CW sources. In order to demonstrate this, a second set of resonators was fabricated on a different active region material^31^, designed to limit self-heating by Joule dissipation through having a lower operating current density and alignment bias. The resonator width was also varied in the 40–45 µm range to explore its effect on the resonant frequency and output power.

**Fig. 5:**
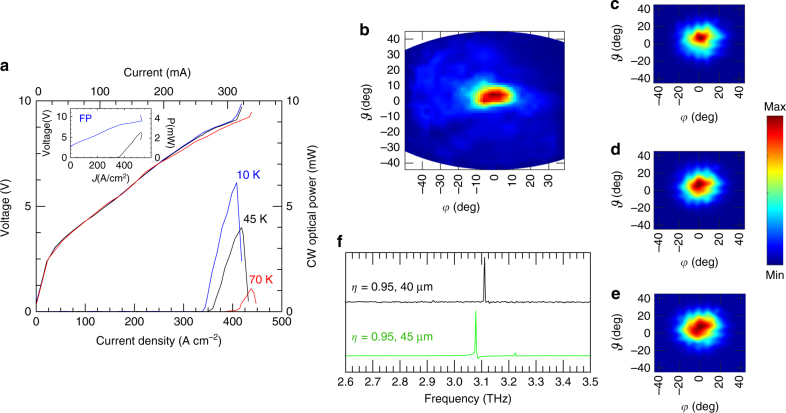
Continuous-wave operation. **a** Voltage–current density (*V–J*) and power–current density (*L–J*) characteristics measured in continuous-wave mode and at different heat-sink temperatures, for a representative wire laser having *η* = 0.95 and an average sinusoidal cavity width of 45 µm. Optical power scales were corrected to account for the detector collection efficiency and the absorption of the polyethylene cryostat window. Inset: *V–J* and *L–J* characteristics measured in continuous-wave mode and at 10 K, for a representative Fabry–Perot (FP) edge-emitting wire laser fabricated from the same semicondictor heterostructure wafer and having a width of 45 µm and a cavity length of 1.1 mm. **b** Far-field intensity pattern of the device in **a**, measured at a distance of 8 cm from the laser surface. **c**–**e** Simulation of the far-field emission intensity profile of the emitting optical mode, derived by applying the Stratton-Chu method to the near-field emission of the fundamental mode, for a hole radius *r* = 5 µm, *r* = 10 µm, and *r* = 12 µm. **f** FTIR emission spectra collected in rapid scan mode at a 0.125 cm^−1^ resolution and with an internal deuterated triglycine sulfate (DTGS) pyroelectric detector for a set of devices having the same *η* = 0.95 and different average widths

Figure 5a shows the *V–J* and *L–J* characteristics of the device with *η* = 0.95 and a width of ~45 µm, driven in CW at different operating temperatures. For comparison the inset of Fig. 5a shows the CW performances of an edge-emitting FP THz QCLs, having comparable dimensions and fabricated from the same semiconductor heterostructure. A maximum CW optical power of 6 mW and a slope efficiency of 100 mW A^−1^ were measured in our sinusoidal wire laser at a heat-sink temperature of 10 K, in a nitrogen-purged atmosphere, against the 2.5 mW of power achieved in the corresponding FP laser. The sinusoidal wire laser reaches maximum WPE in CW of about 0.23%, about 40% larger that that achieved in perfectly phase-matched third-order DFBs^20^. As shown in Fig. 5b, the far-field intensity pattern is characterized by a divergence of Δ*φ* ≈ 8° and Δ*ϑ* ≈ 12°, similar to the previous case. Numerical simulations of the far-field profile (Fig. 5c–e) show that the diameter of the extraction hole array has a role in tailoring properly the optical beam shape: indeed by reducing the hole diameter by 50% (Fig. 5c) the beam shape divergence is slightly reduced in both *φ* and *ϑ* with respect to the selected 10 µm diameter holes array (Fig. 5d); conversely a slight increase of the hole diameter (from 10 to 12 µm, Fig. 5e) starts to significantly deteriorate the optical beam shape.

Figure 5f shows the FTIR emission spectra for two devices with the same extraction parameter (*η* = 0.95), but different sizes. The 40-µm-wide wire laser emits single mode at 3.11 THz, while the larger cavity (45 µm) emits a red-shifted peak at 3.08 THz and a less intense line at 3.22 THz. The spectral emission around 3.1 THz correlates well with the frequency of the lower-edge mode of the fundamental photonic bandgap, as computed in our 3D FEM simulations, which also predict a shift of ≈25 GHz induced by the increased resonator size. In the case of the 45-µm-wide resonator, the low intensity high-frequency peak is very close to the computed frequency of 3.21 THz for the lower-edge mode of the second-order lateral bandgap. The variation of the average ridge width is also reflected in the maximum power output, which is ~10% lower for the 40-µm-wide device with respect to the 45-µm-wide structure. Since the mode has little overlap with the lateral pads and is mostly confined to the 1-mm-long sinusoidal mesa, the extracted optical power consequently scales with the width of the corrugated region. The ridge width also affects the beam collimation in the horizontal direction (*φ* angle), since the 40-µm-wide device has a larger divergence of Δ*φ* ≈ 25°, while in the vertical direction, radiation has a divergence of Δ*ϑ* ≈ 10°, comparable to that of the 45-µm-wide laser with same value of *η* (=0.95).

## Discussion

In conclusion, we have developed a new photonic wire THz resonator architecture exploiting a sinusoidal lateral ridge corrugation as the feedback grating, and a surface array of holes as the extraction grating, the periodicity of which was tuned to optimize the extraction and enhance the output power and laser efficiency. A 42 mW of peak optical power was measured in pulsed regime, with a slope efficiency of ≈250 mW A^−1^. CW operation was also demonstrated with a maximum power output of 6 mW and a maximum WPE of 0.23%. The devices show good beam collimation with a divergence of ~10°. The reached power performances are due to the combined effect of the enhanced coupling losses of our proposed wire laser structure and to the significant beam reshaping which has a positive impact on the collection efficiency.

Optimization of the quantum design would enable the power efficiency to be increased significantly, with no deterioration in the threshold current density and with only a minor influence on the maximum operation temperature, constrained by the lossy absorbing boundary. Possible strategies to improve the present performances furthermore include: the reduction of the area of the lossy absorbing boundary pads to reduce the local lattice temperature, the increase of the wire laser length which plays an important role in increasing the output power and usually reducing the beam divergence^20,22^, the reduction of the thickness of the top highly doped contact layer underlying the un-patterned top metal surface to lower the waveguide losses, and increase the power extraction. Moreover, the use of broader gain region architectures would allow devising an array of on chip wire lasers for tuning the laser emission^32^; furthermore an on-chip mechanical architecture for continuous tuning can also allow adding a further functionality to the system. Remarkably, the proposed architecture is also easily scalable and does not have stringent lithographical constraints—very differently to other photonic engineering approaches reported to date. As such, our devices could become an important underpinning platform for future applications in high-resolution spectroscopy, metrology, and quantum manipulation of cold atoms, for example.

## Methods

### Fabrication

Two different QCL heterostructures were used, both grown by molecular beam epitaxy on an undoped GaAs substrate and consisting of a GaAs/Al_0.15_Ga_0.85_As heterostructure. The first structure (wafer L1023) is based on the three-well resonant-phonon active region design reported in Reference^29^, with layer sequence: **4.3**/8.9/**2.46**/8.15/**4.1**/16 (in nm), where the Al_0.15_Ga_0.85_ barrier layers are indicated in bold, the GaAs wells are in roman, and the underlined figure indicates the presence of a 5-nm-thick Si-doped region in the center of the final well, with a Si doping concentration of 6 × 10^16^ cm^−3^. The second structure (wafer V788) is described in ref. ^31^ and comprises a bound-to-continuum QCL with a single-quantum-well phonon extraction stage. It features a layer sequence of **5.5**/11.0/**1.8**/11.5/**3.8**/9.4/**4.2**/18.4 (in nm), with the last well having a Si doping concentration of 2 × 10^16^ cm^−3^. After growth, a thermo-compressive wafer-bonding technique was performed, bonding the QCL onto an n^+^-GaAs carrier wafer with an Au–Au interface. After selective removal of the host GaAs substrate and an Al_0.5_Ga_0.5_As etch-stop layer, lateral absorbing pads were first defined through the thermal deposition of a thin Cr layer (7 nm), to implement strong absorbing boundary conditions. Then, the sinusoidal top metallization with the extraction hole array was defined by ultra-violet photolithography and a subsequent Cr/Au (5 nm/150 nm) thermal evaporation, partly overlapping the Cr pads. The deposited metal acted as a mask during the inductively coupled plasma reactive ion etching (ICP-RIE) process, so that the n+ top contact layer was selectively etched away only inside the extraction holes but not at the periphery, where the absorbing boundary provided by the Cr and the top contact layer is necessary. Finally, the laser cavity is defined by ICP-RIE etching of the wire laser, ensuring that the lateral corrugation is properly implemented through vertical etching of the semiconductor walls. As a final step, the highly doped GaAs substrate was then lapped down to 150 µm. Individual devices were then indium soldered onto a copper mount and wire-bonded to both lateral pads to guarantee uniform current injection in the device. The FP edge-emitting QCL (from wafer V788) has been fabricated as follows: after a thermo-compressive Au–Au interface wafer-bonding technique of the QCL onto an n^+^-GaAs carrier the host GaAs substrate and the Al_0.5_Ga_0.5_As etch-stop layer, were selectively removed. Then the 45 µm wide laser cavity was defined via ultra-violet photolithography, wet etching and a subsequent top Cr/Au (10 nm/200 nm) thermal evaporation. As a final step, the highly doped GaAs substrate has been lapped down to 150 µm. Individual devices were then indium soldered onto a copper mount and wire-bonded to both lateral pads to guarantee uniform current injection in the device.

### Simulations

A wire cavity having an average width of 40 or 45 µm and a refractive index *n*_eff_ = 3.50 was defined to describe the active material, while an effective refraction index *n*_2_ = 4.43 + 0.31*i* was used to define the lateral contact pads (Fig. 2a). This complex optical constant takes into account both the thin Cr absorbing layer (gray area in Fig. 2a) at the ends of the corrugated mesa and the semiconductor underneath. Both the top and bottom gold metallization layers were modeled as perfect electric conductors. To mimic the propagation of radiation into free space, the resonator is surrounded by an air volume (*n*_air_ = 1) on which scattering boundary conditions are applied.

### Measurement details

The spectra, light–current density (*L–I*) and current density–voltage (*I–V*) characteristics were measured in pulsed mode (typically 2% duty cycle, 200-ns-long pulses) for the devices fabricated from wafer L1023, and in CW for devices fabricated from wafer V194. The spectral characteristics were performed using a FTIR spectrometer, operating in rapid scan mode with a resolution of 0.125 cm^−1^ and an internal deuterated triglycine sulfate (DTGS) detector. The emitted power was measured by a calibrated pyroelectric detector and by a Thomas Keating absolute THz power meter. The far-field emission patterns of the lasers were measured with a pyroelectric detector, having sensitive area of 7 mm^2^, which was scanned on a 8-cm-radius sphere centered on the device surface. The *ϑ* = 0, *φ* = 0 angle corresponds to the direction parallel to the device surface.

### Data availability

The authors declare that the data supporting the findings of this study are available within the paper or are available from the corresponding author upon request.
